# Quality of life in patients with acne in Erbil city

**DOI:** 10.1186/1477-7525-10-60

**Published:** 2012-06-06

**Authors:** Kameran Hassan Ismail, Khalis Bilal Mohammed-Ali

**Affiliations:** 1 Department of Community Medicine, College of Medicine / Hawler Medical University, Erbil, Iraq

**Keywords:** Quality of life, Acne, Erbil

## Abstract

**Background:**

Acne is a very common condition and has a substantial impact on patients’ quality of life. This study was carried out to determine the impact of acne and its clinical severity on health related quality of life in a group of patients attending private clinic in Erbil city, Iraq.

**Methods:**

A cross-sectional study was conducted between July 1^st^, 2011 and November 1^st^, 2011. A convenience sample of 510 patients attending private clinic in Erbil city was taken. Verbal informed consent was obtained from all participants. The Cardiff acne disability index (CADI) was used in this study.

**Results:**

The sample included 510 patients (173 males and 337 females); their mean ± SD age was 20.08 ± 4.23 years (ranged from 11 to 36 years) with a male: female ratio of 0.41:1. The mean ± SD ages of males and females were 18.62 ±3.19 and 20.83 ±4.49 years, respectively (P < 0.001). Results revealed that there is significant association between age and quality of life impairment (P < 0.001), and it revealed that quality of life was more impaired (47.2%) among female than that (37.6%) among male patients (P = 0.038). There was significant association between grading of acne and QOL impairment (P < 0.0001).

**Conclusions:**

Acne negatively affects quality of life, females were more affected than the males, age group 21–25 more than the other age groups and the greater the grade "severity" of acne, the greater the level of impairment of quality of life.

## Introduction

Acne is a common distressing disease that can affect all aspects of an individual’s health-related quality of life (HRQoL), in particular feelings and emotions, personal relationships, sports, social life and employment chances [1]. Acne is a chronic inflammatory disease of the pilosebaceous unit with a multifactorial aetiology. It is one of the most frequent cutaneous diseases, affecting more than 80% of the population at some point in their lives. Although it is generally self-limited a significant percentage of individuals suffer from the disease for years. Independent of its clinical severity, this disease has a great impact on patients’ lives. Acne frequently affects the face, is difficult to hide, and the scars can persist for years or for life. Finally, it is more prevalent in adolescence, a phase of life with great importance in the development of self-confidence and social abilities. Patients with acne have reported functional and emotional effects due to their skin disease comparable to those reported by patients with other diseases. Moreover, when acne was compared with other diseases, acne patients reported levels of social, psychological and emotional problems that were as great as those reported by patients with chronic disabling asthma, epilepsy, diabetes, back pain or arthritis. Therefore, acne is not a trivial disease in comparison with other chronic conditions [2]. There is generally a linear relationship between the clinical severity of acne and impairment of HRQoL, although impairment is also dependent upon a person’s ‘coping ability’. In addition, individuals with little objective evidence of acne may endure severe subjective impairment, greatly affecting their HRQoL [1]. Acne affects many people and can be detrimental to affected patients' quality of life. Assessing the impact of acne on quality of life requires well-validated and reliable measures of acne-specific quality of life that are brief and easy to administer and interpret [3]. Several acne-specific health-related quality of life (HRQoL) instruments now exist, including the Assessment of the psychological and social effects of acne (APSEA), the acne disability index (ADI), the Cardiff acne disability index (CADI) and the dermatology-specific quality of life (DSQL) questionnaire [4]. To our knowledge this is the first cross-sectional study concerning health-related quality of life (HRQoL) of patients with acne in Erbil. This study was carried out to determine the impact of acne and its clinical severity on health related quality of life in a group of patients attending private clinic in Erbil city, Iraq.

## Methods

### Population and data collection

This cross-sectional study conducted between July 1^st^, 2011 and November 1^st^, 2011. A convenience sample included 510 patients (diagnosed as acne) attending private clinic in Erbil city during the study period. Verbal informed consent was obtained from all participants and they were assured that their participations were voluntary and their responses were anonymous and confidential; there were no identifying questions (name) on the questionnaire. The scientific committee of the department of Community Medicine and the ethical committee of College of Medicine at Hawler Medical University approved the study protocol. Necessary corrections were made according to the comments of the committees.

### Instrument

Several instruments exist for measuring skin-related quality of life in dermatology. The Cardiff acne disability index (CADI) was used in this study. The CADI is a validated, brief, acne-specific questionnaire derived from the longer acne disability index has been specifically designed for use in teenagers and young adults with acne. There were five questions relating to the previous month that cover feelings, symptoms, social life and perceived severity. Each question has four possible answers with a maximum of three points and minimum of 0, and a total maximum score of 15. Higher scores indicate more severely affected quality of life [1]. Quality of life according to the CADI scores were categorized into two groups; low scores (<8) and high scores (8+). A short anonymous demographic questionnaire applied which included questions on age, sex, marital status, home ownership, occupation, and years of formal education. The acne were classified according to the type and severity into three categories: mild (few to several papules/pustules), moderate (several to many papules/pustules and few to several nodules), and severe (numerous or extensive papules/pustules and many nodules) [5].

### Data analysis

Data entry and data analysis done by using Statistical package for social sciences (SPSS, version 18.0). P value ≤ 0.05 regarded as statistically significant. Statistical tests included Chi-square (χ^2^) test to compare between the proportions of different “characteristics” among low scores with the same proportions among high scores, and Student t-test to compare between means of numerical variables (age and years of formal education) among low and high scores.

## Results

The sample included 510 patients (173 males and 337 females); their mean ± SD age was 20.08 ± 4.23 years (ranged from 11 to 36 years) with a male: female ratio of 0.41:1. The mean ± SD ages of males and females were 18.62 ±3.19 and 20.83 ±4.49 years, respectively. Table 1 shows that more than half of patients aged 21–25 year have highest score and there was significant association between age and quality of life impairment (P < 0.001).

**Table 1:** Age distribution of patients by QOL scores

**Age (years)**	**Low score (<8)**	**High score (8+)**	**Total**	**χ**^**2**^	**P-value**
	**No. (%)**	**No. (%)**	**No. (%)**		
<16	49 (79.0)	13 (21.0)	62 (100)	21.745	< 0.001
16-20	129 (57.8)	94 (42.2)	223 (100)		
21-25	80 (45.5)	96 (54.5)	176 (100)		
26-30	23 (56.1)	18 (43.9)	41 (100)		
> 30	5 (62.5)	3 (37.5)	8 (100)		
Total	286 (56.1)	224 (43.9)	510 (100)		

Results revealed that QOL was more impaired (47.2%) among female than that (37.6%) among male patients, and there is significant association between sex and QOL scores (P = 0.038). The mean ± SD ages of patients with high score and low scores were 20.86 ±3.89 and 19.47 ±4.38 years, respectively (P < 0.001). There is significant association between grading of acne and QOL impairment (P < 0.0001), as shown in Table 2.

**Table 2:** Description of the sample

**Characteristics**	**Low score (<8)**	**High score (8+)**	**test**	**P-value**
	**No. (%)**	**No. (%)**		
**Sex**				
Male	108 (62.4)	65 (37.6)	4.285*	0.038
Female	178 (52.8)	159 (47.2)		
**Age**				
mean ± SD	19.47 ±4.38	20.86 ±3.89	−3.732**	<0.001
**House ownership**				
Owned	269 (55.6)	215 (44.4)	0.963*	0.326
Rented	17 (65.4)	9 (34.6)		
**Grading**				
Mild	120 (72.7)	45 (27.3)	40.729*	<0.001
Moderate	140 (53.6)	121 (46.4)		
Severe	26 (31.0)	58 (69.0)		
**Occupation**				
Unemployed	243 (57.6)	179 (42.4)	1.91*	0.167
Employed	43 (48.9)	45 (51.1)		
**Years of formal education**				
mean ± SD	9.53 ± 3.97	10.13 ± 4.18	−1.66**	0.097
**Marital status**				
Single	234 (54.4)	196 (45.6)	3.066*	0.08
Married	52 (65.0)	28 (35.0)		
**Address**				
Inside city	209 (53.2)	184 (46.8)	5.84*	0.016
Outside city	77 (65.8)	40 (34.2)		

* Chi square test ** Student t test.

## Discussion

Results revealed that nearly two third of study sample were female which agrees with other studies [6,7]. This doesn't mean that acne is more common in female but it means that girls are more aware about their facial appearance than boys, and they seek for therapy. The QOL is more affected negatively in the age group 21–25 years, and this result agrees with that found in Spain [2] which may reflect the idea that the patients with acne percept that the disease completely disappear after the age of 20, while its existence, continuity, appearing new lesions and the different types of scar tissues as consequences of acne lesions, all were opposite of his/her perception. In addition getting more mature in his life making the feeling and sensitivity and the cumulative negative reflection of the community about his/her condition may be another factor, also the idea of marriage in this age group in Erbil society may be another factor increasing the impact on his feeling. In the current study, the females' QOL were significantly more affected than that of males, as agrees with other studies, [2,8] and this may be due to the fact that the girls are more sensitive and more influenced about their appearance in the community. However this finding disagrees with findings in Mersin and Egypt [9,10]. In the current study the owner of house as an economic factor hasn't significant effect on degree of impairment of quality of life which may explain the equality of importance of face appearance among both rich and poor people.

Results also revealed that the QOL was more affected, as the severity (grading) of the lesion were increasing, which agrees with that seen in Turkey [9] and France [11] which may be logic finding that how much the lesions increased in severity, the awareness, sensitivity, negative feeling and anxiety will increase. The current study showed that the degree of impairment of quality of life is not affected by the type of occupation, which may mean that what ever is the occupation the appearance of face has its importance in the community, and the quality of life was not affected by the levels of education which was the same finding in Greece [12], meaning that every body is aware of his appearance, whether he is educated or not, and the recognition of ugly appearance of the people doesn't require a high level of education. The marital status of patients was not a significant factor on degree of impairment of quality of life which may be need of both married and unmarried people to a good look, and it may be all the study population are young age population, and their general health condition is good and lack other health problems, so it will be a priority health problem for them a clear looking face. The current study showed that QOL of urban people were more affected than that of rural which is consistent with findings in India, it may be explained by many factors; the surrounding environment, pattern of life in cities, priorities of the community, concern of the people about their skin appearances and the larger amount of people daily facing are different from that of rural areas [13].

Because the venue of this study is a private clinic and not a hospital, it may be a barrier for patients with low socioeconomic status to consult and ask for treatment. Despite this limitation, this is the first study to be achieved in the study setting regarding both QOL of patients with skin diseases in general and acne patients specifically, and it can be used as a solid database for future studies about QOL of patients with skin diseases.

## Conclusions

Acne negatively affects quality of life. Females were more affected than males, age group 21–25 more affected than the other age groups and the greater the grade "severity" of acne, the greater the level of impairment of quality of life. The impact of acne on quality of life can be measured using general health measures, dermatology-specific measures or acne-specific measures. In order for quality of life measures to be used more frequently in the routine clinical work, they need to be easy to use, the scores need to be meaningful and they need to be readily accessible. Clinicians must be convinced that the information gained from using them is of benefit in guiding them to make optimum clinical decisions for their patients, and they need to become aware that the use of these measures may help to justify their clinical decisions. Quality of life measures can influence the choice of therapy. In patients with a severe impact on their quality of life, a more aggressive therapy may be justified.

## Competing interests

The authors declare that they have no competing interests.

## Authors’ contributions

IKH and M-AKB participated in designing the study. IKH drafted the first version of the paper and carried out the data analysis, designing tables and reviewing the article and references. M-AKB carried out the patients’ interview, wrote the discussion section, and reviewing the article. All authors read and approved the final manuscript.
